# Oral Anticoagulation and Risk of Symptomatic Hemorrhagic Transformation in Stroke Patients Treated With Mechanical Thrombectomy: Data From the Nordictus Registry

**DOI:** 10.3389/fneur.2020.594251

**Published:** 2020-11-26

**Authors:** María E. Ramos-Araque, Alba Chavarría-Miranda, Beatriz Gómez-Vicente, Elena López-Cancio Martínez, María Castañón Apilánez, Mar Castellanos, María López Fernández, Herbert Tejada Meza, Javier Marta Moreno, Javier Tejada García, Iria Beltrán Rodríguez, Patricia de la Riva, Noemi Díez, Susana Arias Rivas, María Santamaría Cadavid, Yolanda Bravo Anguiano, Mónica Bártulos Iglesias, Enrique Jesús Palacio Portilla, Marian Revilla García, Juan José Timiraos Fernández, Naroa Arenaza Basterrechea, José Luis Maciñeiras Montero, Pablo Vicente Alba, Francisco José Julián Villaverde, Ana Pinedo Brochado, Itxaso Azkune, Freijo M. Mar, Alain Luna, Juan F. Arenillas

**Affiliations:** ^1^Department of Neurology, Institute of Biomedical Research of Salamanca, Hospital Universitario de Salamanca, Salamanca, Spain; ^2^Department of Neurology, Hospital Clínico Universitario de Valladolid, Valladolid, Spain; ^3^Department of Neurology, Stroke Unit, Hospital Universitario Central de Asturias, Oviedo, Spain; ^4^A Coruña Biomedical Research Institute/A Coruña University Hospital, A Coruña, Spain; ^5^Department of Neurology, Stroke Unit, Hospital Universitario Miguel Servet, Zaragoza, Spain; ^6^Department of Radiology, Neurointerventionism Unit, Hospital Universitario Miguel Servet, Zaragoza, Spain; ^7^Instituto de Investigación Sanitaria de Aragón (IIS Aragón), Zaragoza, Spain; ^8^Department of Neurology, Complejo Asistencial Universitario de León, León, Spain; ^9^Department of Neurology, Donostia University Hospital, San Sebastián, Spain; ^10^Department of Neurology, Complejo Hospitalario Universitario de Santiago, Santiago de Compostela, Spain; ^11^Department of Neurology, Hospital Universitario de Burgos, Burgos, Spain; ^12^Department of Neurology, Hospital Universitario Marqués de Valdecilla, Santander, Spain; ^13^Department of Neurology, Stroke Unit, Hospital Universitario de Araba, Vitoria, Spain; ^14^Department of Neurology, Complejo Hospitalario Universitario de Vigo, Vigo, Spain; ^15^Department of Neurology, Hospital San Pedro, La Rioja, Spain; ^16^Department of Neurology, Hospital Galdakao-Usansolo, Bizkaia, Spain; ^17^Neurovascular Group, Neurology Department, Biocruces Research Health Institute, Cruces Hospital, Barakaldo, Spain; ^18^Clinical Neurosciences Research Group, Department of Medicine, University of Valladolid, Valladolid, Spain

**Keywords:** anticoagulants, stroke, thrombectomy, intracranial hemorrhage, vitamin K

## Abstract

**Introduction:** We aimed to evaluate if prior oral anticoagulation (OAC) and its type determines a greater risk of symptomatic hemorrhagic transformation in patients with acute ischemic stroke (AIS) subjected to mechanical thrombectomy.

**Materials and Methods:** Consecutive patients with AIS included in the prospective reperfusion registry NORDICTUS, a network of tertiary stroke centers in Northern Spain, from January 2017 to December 2019 were included. Prior use of oral anticoagulants, baseline variables, and international normalized ratio (INR) on admission were recorded. Symptomatic intracranial hemorrhage (sICH) was the primary outcome measure. Secondary outcome was the relation between INR and sICH, and we evaluated mortality and functional outcome at 3 months by modified Rankin scale. We compared patients with and without previous OAC and also considered the type of oral anticoagulants.

**Results:** About 1.455 AIS patients were included, of whom 274 (19%) were on OAC, 193 (70%) on vitamin K antagonists (VKA), and 81 (30%) on direct oral anticoagulants (DOACs). Anticoagulated patients were older and had more comorbidities. Eighty-one (5.6%) developed sICH, which was more frequent in the VKA group, but not in DOAC group. OAC with VKA emerged as a predictor of sICH in a multivariate regression model (OR, 1.89 [95% CI, 1.01–3.51], *p* = 0.04) and was not related to INR level on admission. Prior VKA use was not associated with worse outcome in the multivariate regression model nor with mortality at 3 months.

**Conclusions:** OAC with VKA, but not with DOACs, was an independent predictor of sICH after mechanical thrombectomy. This excess risk was associated neither with INR value by the time thrombectomy was performed, nor with a worse functional outcome or mortality at 3 months.

## Introduction

Symptomatic intracranial hemorrhage (sICH) is a life-threatening complication associated with reperfusion therapies after acute ischemic stroke (AIS) (1). Treatment with direct oral anticoagulants (DOACs), or vitamin K antagonists (VKA) with an international normalized ratio (INR) > 1.7 was a contraindication for the administration of intravenous thrombolysis due to the increased risk of intracranial bleeding (2). However, the risk of developing symptomatic hemorrhagic transformation in anticoagulated patients is not yet fully established after endovascular treatment (EVT) (3). Patients taking oral anticoagulation (OAC) were often underrepresented in pivotal clinical trials of mechanical thrombectomy, accounting for <5% of the total (4). To date, it has been a matter of controversy if EVT in AIS with prior anticoagulant treatment carries a major risk of bleeding. It is essential to select the best therapeutic approach in this subset of patients, because despite best medical treatment with anticoagulation 1–3% of patients will develop strokes annually (5).

In addition, there is little data regarding the possible influence of the type of anticoagulant (VKA vs. DOACs) and the risk of sICH after EVT in AIS. Recently, a systematic review and meta-analysis demonstrated that patients under VKA treatment had an increased risk of sICH and mortality after mechanical thrombectomy (1), although another observational study did not report differences in the rates of sICH between patients under VKA compared with DOACs (6).

Data are also limited and inconclusive regarding the relationship between the intensity of anticoagulation and the risk of sICH after EVT (7, 8). To date, neither INR level above a certain threshold nor DOAC activity values have been associated with an increased sICH or poor outcome risk. Thus, clarification of these scientific questions would satisfy considerable clinical need, since EVT is the only therapeutic option for anticoagulated AIS patients with a large vessel occlusion (9).

We aimed to study whether prior OAC, either assessed as a whole group or focusing on the type of oral anticoagulants used, was associated with an increased risk of sICH in AIS patients. As secondary aims, we studied the relationship between INR level at the time of treatment and sICH risk and the impact of prior OAC on functional outcome and predictors of mortality in all cohort.

## Materials and Methods

### Study Design and Patient Selection

We performed a retrospective, observational, multicentric study based on a prospective registry of all consecutive AIS patients treated with reperfusion therapies in tertiary Stroke Centers in Spain included in the NORDICTUS Registry between 2017 and 2019. NORDICTUS is a research network in cerebrovascular diseases that brings together all public hospitals with stroke units in North-West Spain serving an area of 11.5 million people. Fourteen tertiary stroke centers participated in the investigation and the study was approved by their respective local institutional Clinical Research Ethics Committee. Written informed consent was obtained from all included patients or their relatives giving permission to enter their information into our reperfusion registry and to use of the data for scientific purposes, in accordance with the Spanish Personal Data Protection law.

Patients treated with mechanical thrombectomy included in the Nordictus Registry were selected for this study if they fulfilled the following criteria: (1) patients with disabling focal neurological deficit and ischemic stroke with demonstrated vessel occlusion, (2) time from symptom onset to groin puncture <24 h, including wake up strokes and unknown onset, (3) no intracranial hemorrhage at baseline cranial tomography (CT); (4) no extensive early ischemic signs as defined by an Alberta Stroke Program Early CT Score (ASPECTS) > 5 (10); (5) in patients with time from symptom onset to EVT >6 h, presence of a target mismatch profile on CT perfusion (11); and (6) absence of previous relevant disability evaluated by the modified Rankin Scale (mRS) (pre-stroke mRS score ≤ 2). If no contraindication existed (including INR <1.7 in those under VKA or DOAC in the prior 48 h), prior treatment with rt-PA was administered before EVT following regular guidelines.

### Clinical Data and Baseline Variables

All patients included were clinically managed according to our institutional protocols, which are based on current international stroke guidelines. Our reperfusion registry includes the following baseline variables: age, sex, previous functional disability defined by mRS, information about the presence of arterial hypertension, diabetes, dyslipidemia, smoking, atrial fibrillation, history of coronary disease, or stroke prior to admission, previous treatment with antiplatelets, or anticoagulants and their type (VKA, DOACs, heparin). Anticoagulation status was recorded at the time of admission, and urgent INR was obtained in all patients prior to thrombectomy. Information regarding baseline clinical stroke severity according to National institute of Health Stroke Scale (NIHSS) score, location of artery occlusion and treatment, type of anesthesia during the procedure, and time intervals were also recorded. Stroke subtypes were categorized according to the Trial of ORG 10172 in Acute Stroke Treatment (TOAST) classification (12).

### Neuroimaging Protocol

A basal cranial noncontrast CT and CT angiography (CTA) scan was performed before endovascular treatment. Extent of early ischemic changes was evaluated by ASPECTS, and the arterial occlusion site was defined based on the CTA.

Target mismatch was established by a core volume of <70 cm^3^, a penumbra/core ratio >1.8, and a mismatch volume >15 ml.

Recanalization after EVT was evaluated by the Thrombolysis in Cerebral Infarction (TICI) scale (13) and considered successful when TICI 2b-3 was achieved after the procedure.

A follow-up brain CT was performed after 24 h of treatment or earlier in case clinical deterioration occurred with the intention to assess the presence of hemorrhagic transformation. Hemorrhagic transformation subtypes were categorized according to the European Cooperative Acute Stroke Study (ECASS-2) definition as hemorrhagic infarction (HI) types 1 and 2 and parenchymal hematoma types 1 and 2 (14). In addition, sICH was defined according to Heidelberg criteria (15) as hemorrhagic transformation that was associated with significant neurological worsening, which was defined as an increase of four or more points in the NIHSS scale (15).

### Primary and Secondary Outcome

The primary study outcome was the presence of sICH after endovascular treatment.

As secondary outcomes, we aimed to evaluate whether the INR value in patients on VKA at the time of treatment carries a higher risk of sICH. We also studied predictors of mortality and poor functional outcome at 3 months after the stroke, which was defined as a modified Rankin score >2.

Also, we compared outcomes in patients with and without previous use of OAC and according to the type of oral anticoagulants (VKA vs. DOACs).

### Statistical Analysis

Data from each center were prospectively included in an anonymized online database of NORDICTUS research groups and statistical analysis after exporting the data was performed using SPSS statistics version 24 (Chicago, Illinois, USA).

Baseline quantitative continuous variables were expressed using their mean, standard deviation (SD) or median (interquartile range), as appropriate. Categorical variables were presented as number of cases and their percentage (%). Normality distribution of the data was assessed by using Kolmogorov-Smirnov test. Baseline characteristics of the study groups were compared using the Chi-square test for discrete variables, t-student test for quantitative variables with a normal distribution and Mann-Whitney U test for quantitative variables not following a normal distribution.

Bivariate analyses were performed to detect baseline variables associated with the occurrence of the primary and secondary endpoints. A logistic regression analysis was performed to identify independent predictors of sICH, poor functional outcome, and mortality. Adjustment was performed for all variables showing a *p* < 0.1 on the respective bivariate analysis. Age, arterial hypertension, dyslipidemia, and atrial fibrillation were also entered into the model as relevant potential confounding factors derived from recent literature (6) and from our bivariate analysis of variables associated with prior OAC. We performed a subgroup analysis to evaluate the influence of the type of anticoagulant (VKA vs. DOACs) on the outcomes. The logistic regression data are presented as adjusted odds ratio (OR) and respective 95% confidence intervals (CI) and statistical significance level was defined as a *p* value < 0.05. OR for ASPECTS and mRS was expressed as per 1-point increased in LR model. In the subgroup of VKA-treated patients, we performed a multivariate analysis to analyze the relationship between pretreatment INR level and sICH risk implementing continuous INR.

## Results

From January 2017 to December 2019, 1,710 consecutive patients treated with mechanical thrombectomy were included in the NORDICTUS stroke registry. From those patients who underwent EVT, 1,455 fulfilled our inclusion criteria. Reasons for exclusion of the remaining patients were treatment with heparin (*n* = 14), prestroke mRS >2 (*n* = 172), and unavailable follow-up data (*n* = 69).

The distribution of baseline variables of the study group is shown in Table 1. Six hundred and sixty-four patients (46%) were women, the mean age of the overall population was 72.42 years (SD: 12.84) and median NIHSS at admission was 16 (10–20). Two hundred and seventy-four patients (19%) were on oral anticoagulants, 193 on VKA (70%) and 81 (30%) on DOACs.

**Table 1:** Baseline characteristics of whole study sample and bivariate analysis according to OAC status.

	**All cohort (*n* = 1,455)**	**Non-OAC (*n* = 1,181)**	**DOACs (*n* = 81)**	**VKA (*n* = 193)**	***p* Value^**a**^**	***P* value^**b**^**
Age (year, mean ± SD)	72.4 ± 12.8	71.5 ± 13.2	76.37 ± 9.79	76.66 ± 10.2	<0.001	0.73
Sex [women; *n* (%)]	664 (46)	525 (45)	38 (48)	101 (52)	0.13	0.50
Hypertension [*n* (%)]	927 (64)	729 (62)	59 (73)	139 (72)	0.005	0.53
Diabetes mellitus [*n* (%)]	285 (20)	221 (19)	19 (24)	45 (23)	0.22	1
Dyslipidemia [*n* (%)]	705 (49)	550 (47)	47 (58)	108 (57)	0.01	0.89
Atrial fibrillation [*n* (%)]	402 (28)	153 (13)	78 (96)	171 (89)	<0.001	0.11
History of coronary disease [*n* (%)]	197 (14)	140 (12)	17 (21)	40 (21)	<0.001	1
Previous stroke [*n* (%)]	188 (13)	121 (10)	18 (23)	49 (25)	<0.001	0.64
Smoking [current or past; *n* (%)]	463 (33)	398 (35)	19 (24)	46 (24)	<0.001	0.90
Prior mRS [0–1; *n* (%)]	1,282 (88)	1,065 (90)	60 (74)	157 (81)	<0.001	0.38
Antiplatelet treatment	343 (24)	323 (27)	5 (6)	15 (8)	<0.001	0.59
Acetylsalicylic acid [*n* (%)]	343 (24)	262 (22)	3 (4)	12 (6)	<0.001	0.56
Clopidogrel [*n* (%)]	277 (19)	33 (3)	2 (3)	2 (1)	0.35	0.58
Double antiplatelet therapy [*n* (%)]	37 (3)	17 (1)	0	1 (1)	0.32	1
Baseline NIHSS median (IQR)	16 (10–20)	16 (10–20)	15 (9–20)	18 (12–21)	0.027	0.05
Baseline ASPECTS median (IQR)	9 (8–10)	9 (8–10)	10 (8–10)	8 (7–10)	0.13	<0.001
MCA-M1 occlusion [*n* (%)]	792 (55)	625 (54)	49 (61)	118 (62)	0.09	0.34
Tandem occlusion [*n* (%)]	250 (17)	230 (20)	6 (7)	14 (7)	<0.001	1
TOAST [cardioembolic origin; *n* (%)]	682 (50)	438 (40)	70 (91)	174 (93)	<0.001	0.58
Known time of symptom onset (%)	964 (66)	785 (67)	53 (66)	126 (65)	0.94	1
**Time intervals [median (IQR) min]**						
Onset-to-door time	115 (228–61)	118 (234–61)	97 (54–236)	104 (60–216)	0.15	0.73
Onset-to-arterial puncture	218 (318–161)	225 (320–165)	197 (152–300)	201 (160–312)	0.07	0.30

*OAC, oral anticoagulation; DOACs, direct oral anticoagulants; VKA, vitamin K antagonist; NIHSS, National Institute of Health Stroke Scale; TOAST, Trial of Org 10172 in acute stroke registry; ASPECTS, Alberta Stroke Program Early CT Score; MCA, middle cerebral artery; IQR, interquartile range; mRS, modified Rankin Scale*.

a *p-values indicate comparisons between overall groups*.

b *p-values indicate comparisons between DOACs and VKA*.

Regarding baseline characteristics according to anticoagulation status (Table 1), patients on OAC (DOACs and VKA) were older and had significantly higher prevalence of arterial hypertension, dyslipidemia, previous atrial fibrillation, ischemic cardiopathy, and previous stroke. VKA patients had higher punctuations on baseline NIHSS and lower ASPECTS compared with DOACs and non-OAC patients. Site of occlusion were not statistically different between both groups, except for a lower proportion of tandem occlusion in the OAC group. Prestroke functional status (mRS 0–1) was worse compared with patients who were not on oral anticoagulants. Also, patients on OAC tended to have more cardioembolic strokes.

In terms of safety, the proportion of patients with sICH was similar in patients with and without oral anticoagulants.

As shown in Supplementary Table 1, the proportion of patients treated with rt-PA differed between two groups. As expected, prior intravenous thrombolysis was less frequently given in the OAC group (*p* = <0.001). Successful and near to complete recanalization (TICI 2b or 3) was achieved in 86% patients of the overall sample and was similar in non-OAC vs. OAC patients.

### Main Outcome: Symptomatic Intracerebral Hemorrhage

Bivariate analysis of baseline variables potentially associated with sICH is shown in Table 2. Patients who had a sICH were more often on VKA treatment, had worse prestroke functional status, and less often had a more proximal large vessel occlusion. Higher baseline NIHSS, lower ASPECT score, and longer onset to door and onset to groin puncture times were also associated with a higher sICH risk on bivariate analysis.

**Table 2:** Baseline characteristics of whole study sample and bivariate analysis according to symptomatic intracranial hemorrhage.

	**No sICH (*n* = 1,374)**	**sICH (*n* = 81)**	***p*-value**
Age (year, mean ± SD)	72.3 ± 12.9	73.4 ± 11.1	0.78
Sex [women; *n* (%)]	631 (46)	33 (42)	0.48
Hypertension [*n* (%)]	874 (64)	53 (65)	0.81
Diabetes mellitus [*n* (%)]	266 (20)	19 (24)	0.38
Dyslipidemia [*n* (%)]	668 (49)	37 (46)	0.64
Atrial fibrillation [*n* (%)]	376 (28)	26 (33)	0.47
History of coronary disease [*n* (%)]	187 (14)	10 (12)	0.86
Previous Stroke [*n* (%)]	172 (13)	16 (20)	0.08
Smoking (current or past) [*n* (%)]	437 (33)	26 (32)	0.49
Prior mRS 0–1 [*n* (%)]	1218 (89)	64 (79)	0.01
OAC [*n* (%)]	254 (19)	20 (25)	0.18
Treatment with VKA	174 (13)	19 (24)	0.01
Treatment with DOACs	80 (6)	1 (1.2)	0.08
Antiplatelet [any, *n* (%)]	329 (24)	14 (17)	0.5
Acetylsalicylic acid [*n* (%)]	264 (19)	13 (16)	0.56
Clopidogrel [*n* (%)]	36 (3)	1 (1)	0.7
Double antiplatelet therapy [*n* (%)]	18 (1)	0	0.61
Baseline NIHSS [median, (IQR)]	16 (10–20)	18 (13–21)	0.02
Baseline ASPECTS [median, (IQR)]	9 (8–10)	8 (9–7)	<0.001
MCA-M1 occlusion [*n* (%)]	755 (56)	37 (46)	0.04
Tandem occlusion [*n* (%)]	232 (17)	18 (22)	0.22
TOAST [cardioembolic origin; *n* (%)]	649 (50)	33 (45)	0.81
Known time of symptom onset (%)	912 (66)	52 (64)	0.71
**Time intervals [median (IQR) min]**			
Onset-to-door time	110 (60–224)	164 (90–265)	<0.001
Onset-to-arterial puncture	214 (160–315)	278 (217–356)	<0.001

*NIHSS, National Institute of Health Stroke Scale; TOAST, Trial of Org 10172 in acute stroke registry; ASPECTS, Alberta Stroke Program Early CT Score; MCA, middle cerebral artery; IQR, interquartile range; mRS, modified Rankin Scale; OAC, oral anticoagulation; DOACs, direct oral anticoagulants; VKA, vitamin K antagonist*.

After multivariate analysis, prior VKA treatment (OR, 1.89 [95% CI, 1.01–3.51], *p* = 0.04), prior mRS (OR, 1.43 [95% CI, 1.03–1.99]), *p* = 0.03), and basal ASPECTS on admission (OR, 0.72 [95% CI, 0.61–0.84]), *p* < 0.001) emerged as independent predictors of sICH (Table 3).

**Table 3:** Logistic regression: predictors of symptomatic intracranial hemorrhage.

	**OR (IC 95%)**	***p*-value**
Prior mRS	1.43 (1.03–1.99)	0.03
Baseline NIHSS	1 (0.96–1.05)	0.72
ASPECTS	0.71 (0.61–0.84)	<0.001
Treatment with VKA	1.89 (1.01–3.51)	0.04
Treatment with DOACs	0.32 (0.04–2.42)	0.27
Vessel occlusion MCA-M1	0.91 (0.71–1.16)	0.46
Age	1.01 (0.99–1.04)	0.2
Arterial Hypertension	0.89 (0.51–1.55)	0.68
Hypercholesterolemia	0.82 (0.49–1.37)	0.46
Atrial fibrillation	0.79 (0.54–1.17)	0.25

*mRS, modified Rankin Scale; NIHSS, National Institute of Health Stroke Scale; ASPECTS, Alberta Stroke Program Early CT Score; MCA, middle cerebral artery; VKA, vitamin K antagonist*.

### Secondary Outcomes

In the group of patients on VKA, we evaluated the association between pre-treatment INR and sICH risk using INR as a continuous variable. The multivariate analysis did not disclose any significant association between these variables in an unadjusted (OR, 1.13 [95% CI, 0.52–2.46], *p* = 0.74) and after adjusted model (OR, 1.01 [95% CI, 0.42–2.41], *p* = 0.97).

Patients on oral anticoagulants had lower rates of functional independence at the third month (50 vs. 43%, *p* = 0.050). No significant differences in mortality within 90 days were observed in patients under oral anticoagulant treatment vs. non-anticoagulated patients.

Prior VKA treatment was associated with a worse outcome in bivariate analysis (*p* = 0.002) but not after multivariate adjustment (OR, 1.13 [95% CI, 0.76–1.67], *p* = 0.52) (Table 4).

**Table 4:** Logistic regression model: predictors of poor functional outcome at 3 months of all cohort.

	**OR (IC 95%)**	***p*-value**
Age	1.04 (1.02–1.05)	<0.001
Sex (women)	1 (0.75–1.32)	0.99
Arterial hypertension	1.20 (0.89–1.60)	0.21
Diabetes mellitus	1.21 (0.86–1.70)	0.26
Prior mRS	1.61 (1.31–1.98)	<0.001
Baseline NIHSS	1.09 (1.07–1.11)	<0.001
Vessel occlusion MCA-M1	1.06 (0.95–1.17)	0.27
VKA treatment	1.13 (0.76–1.67)	0.52
ASPECTS	0.73 (0.66–0.81)	<0.001
Atrial fibrillation	0.92 (0.77–1.10)	0.39
Onset-to-door time	1 (0.99–1)	0.68

*mRS, modified Rankin Scale; NIHSS, National Institute of Health Stroke Scale; MCA, middle cerebral artery; VKA, vitamin K antagonist; ASPECTS, Alberta Stroke Program Early CT Score*.

As shown in Supplementary Table 2, after multivariate analysis, the predictors of mortality in all cohort were older age (OR, 1.03 [95% CI, 1.02–1.05], *p* < 0.001), worse prestroke functional status (OR, 1.37 [95% CI, 1.12–1.68], *p* = 0.002), higher punctuation on NIHSS on admission (OR, 1.07 [95% CI, 1.05–1.01], *p* < 0.001), and higher rates of proximal occlusion (OR, 1.11 [95% CI, 1–1.23], *p* = 0.044).

### Subgroup Analysis in OAC Patients (VKA vs. DOACs)

We performed a separate multivariate-adjusted logistic regression model in the group of patients treated with oral anticoagulants. Prior anticoagulation with VKA was associated with a significant increase in sICH risk when compared with DOACs (OR, 8.41 [95% CI, 1.03–68.54]), *p* = 0.04).

## Discussion

In this observational study based on a prospective multicenter reperfusion registry, the most important finding was that prior anticoagulation with VKA emerged as an independent predictor of sICH in AIS patients undergoing EVT. This excess bleeding risk associated with VKA contrasts with the lack of association between the use of DOACs and sICH risk, which suggests that DOACs have a better safety profile than VKA in the setting of EVT for AIS. This different safety profile is another solid reason to prescribe DOACs over VKA to try to prevent ischemic stroke in patients with non-valvular atrial fibrillation.

Our main observation is in line with a recent systematic review and meta-analysis (1), that has reported higher sICH rates in VKA users but not in patients under DOACs. The authors of that study described a significant increase in mortality in patients anticoagulated with VKA, which we did not find in our series. This excess mortality associated with VKA has also been found by other studies. Taken together, all these findings contribute to raise the questions of whether there is a high-bleeding risk profile among patients treated with VKA undergoing EVT and if EVT is safe in all circumstances for patients anticoagulated with VKA. Future research should address these issues (1, 16). Use of VKA was associated with worse functional outcome at 3 months in bivariate analysis but did not predict functional dependence in the multivariate regression model, in accordance with a recent study (6). This lack of association with poor functional outcome could be due to the role of confounding factors associated with the use of VKA, such as age, comorbidities, premorbid status, or higher incidence of atrial fibrillation (17) in patients under VKA treatment compared with nonanticoagulated patients.

In our study, we did not find an association between pre-EVT INR level and the risk of sICH among VKA patients. However, only seven patients had a value of INR >3, so this precludes any conclusion. On the other hand, our result is in agreement with previous works showing that increased INR was not associated with higher sICH risk (6–8, 18). Thus, pretreatment INR level may not reflect the risk of bleeding after EVT, and therefore exclusion of EVT candidates based on INR level, as done with intravenous thrombolysis patients, does not seem to be justified according to existing evidence. The fact that these patients had an ischemic stroke under anticoagulation and subsequently a severe hemorrhagic complication after EVT, might be related to high instability or fluctuations of the intensity of anticoagulation under VKA (3). In this setting, it would have been ideal to evaluate the risk associated with the percentage of time patients were in range of effective anticoagulation during the previous months, but this data was not available in our registry.

In our study, the rates of sICH between oral anticoagulated patients and those not taking oral anticoagulants was similar, which is also in agreement with previous reported series (18, 19). Moreover, recanalization rates were also similar in the two groups. Other series have reported better TICI scores in patients receiving OAC, raising the prospect that anticoagulation itself may reduce fibrin formation and facilitate EVT (6) or that thrombus from cardioembolic origin could be more prone to be removed (20), but this is unclear (21). According to our results, we can affirm that EVT is safe and feasible in the context of previous anticoagulation as previously reported (22). Our proportion of anticoagulated patients subjected to EVT (19%) is slightly higher than rates reported in most recently published data (6); this emphasizes the importance of the occurrence of stroke in previous anticoagulated patients who will further require reperfusion therapy (17). Our rate of sICH is 5.6%, which is also in line with the proportion reported in recent series of patients with AIS treated with mechanical thrombectomy (1, 6). Even with this higher risk of sICH in VKA patients, EVT should be performed if there are no other contraindications. Moreover, we have to identify a clinical profile that can predict the higher risk of bleeding, to determine if EVT is safe in all circumstances for VKA patients.

This study faces some limitations. First, this is a retrospective analysis from non-randomized, observational data. Second, the selection of patients for EVT was conducted at the discretion of each center and this could represent a confounding factor for the indication of EVT. Third, we could not estimate DOACs compliance or activity or INR therapeutic range of VKA patients, which perhaps facilitates the risk of sICH after EVT, even when IVT was withheld in 91% of OAC patients in our series. Finally, the small number of sICH events limited the number of variables entered in the multivariate regression model. Therefore, the influence of other nonconsidered potential confounding variables cannot be entirely ruled out.

## Conclusions

Prior use of VKA, but not of DOACs, was associated with an increased risk of sICH in patients with AIS subjected to EVT. However, this was not related to worse outcome at 3 months. The risk of sICH was not associated with the intensity of anticoagulation as determined by the pre-EVT INR level in patients treated with VKA. Prior anticoagulation with DOACs appears to have a better safety profile when compared with VKAs in AIS patients undergoing thrombectomy.

## Data Availability Statement

The datasheets are available upon reasonable request to the corresponding author.

## Ethics Statement

The studies involving human participants were reviewed and approved by fourteen tertiary stroke centers participated in the investigation and the study was approved by their respective local institutional Clinical Research Ethics Committee. The patients/participants provided their written informed consent to participate in this study.

## Author Contributions

MER-A drafted the manuscript and performed statistical analysis. AC-M partly drafted the manuscript. EL-CM, MC, JMar, JTG, PR, and EPP critically reviewed the manuscript. AC-M, BG-V, EL-CM, MCA, MC, MLF, HTM, JMar, JTG, IBR, PR, ND, SAR, MSC, YBA, MBI, MRG, JTF, NAB, JMac, PVA, FJV, APB, IA, FM, and AL acquired data. JFA conceived the study, acquired data, and critically reviewed the manuscript. All authors contributed to the article and approved the submitted version.

## Conflict of Interest

JA reports having received speaker or consultant honoraria by: BI, Bayer, Daiichy Sankyo, Pfizer, Amgen, Medtronic. MC has received speaker honoraria from Pfizer-BristolMyers Squibb, Daichii-Sankyo, and Boehringer-Ingelheim, and for providing consultancy on advisory board meetings from Amgen and Allergan. The remaining authors declare that the research was conducted in the absence of any commercial or financial relationships that could be construed as a potential conflict of interest.

## References

[1] Meinel TR Kniepert JU Seiffge DJ Gralla J Jung S Auer E. Endovascular stroke treatment and risk of intracranial hemorrhage in anticoagulated patients. Stroke. (2020) 51:892–8. 10.1161/STROKEAHA.119.026606 31992179

[2] Powers WJ Rabinstein AA Ackerson T Adeoye OM Bambakidis NC Becker K. Guidelines for the early management of patients with acute ischemic stroke: 2019 update to the 2018 guidelines for the early management of acute ischemic stroke a guideline for healthcare professionals from the American Heart Association/American Stroke A. Stroke. (2019) 50:344–418. 10.1161/STR.0000000000000211 31662037

[3] L'allinec V Ernest M Sevin-Allouet M Testard N Delasalle-Guyomarch B Guillon B. Safety and efficacy of mechanical thrombectomy in acute ischemic stroke of anticoagulated patients. J Neurointerv Surg. (2018) 10:E29. 10.1136/neurintsurg-2017-013714 29602862

[4] Vidale S Agostoni E. Endovascular treatment of ischemic stroke: an updated meta-analysis of efficacy and safety. Vasc Endovascular Surg. (2017) 51:215–9. 10.1177/1538574417698905 28424039

[5] Wong JWP Churilov L Dowling R Mitchell P Bush S Kanesan L. Safety of endovascular thrombectomy for acute ischaemic stroke in anticoagulated patients ineligible for intravenous thrombolysis. Cerebrovasc Dis. (2019) 46:193–9. 10.1159/000493801 30384367

[6] Goldhoorn R-JB van de Graaf RA van Rees JM Lingsma HF Dippel DWJ Hinsenveld WH. Endovascular treatment for acute ischemic stroke in patients on oral anticoagulants. Stroke. (2020) 51:1781–9. 10.1161/STROKEAHA.119.028675 32390550

[7] Mundiyanapurath S Tillmann A Möhlenbruch MA Bendszus M Ringleb PA. Endovascular stroke therapy may be safe in patients with elevated international normalized ratio. J Neurointerv Surg. (2017) 9:1187–90. 10.1136/neurintsurg-2016-012757 27856650

[8] Pandhi A Tsivgoulis G Ishfaq MF Katsanos A Magoufis G Malhotra K. Mechanical thrombectomy outcomes in large vessel stroke with high international normalized ratio. J Neurol Sci. (2019) 396:193–8. 10.1016/j.jns.2018.11.019 30481657

[9] Diener HC Foerch C Riess H Röther J Schroth G Weber R Treatment of acute ischaemic stroke with thrombolysis or thrombectomy in patients receiving anti-thrombotic treatment. Lancet Neurol. (2013) 12:677–88. 10.1016/S1474-4422(13)70101-7 23726849

[10] Pexman JH Barber PA Hill MD Sevick RJ Demchuk AM Hudon ME. Use of the Alberta Stroke Program Early CT Score (ASPECTS) for assessing CT scans in patients with acute stroke. AJNR Am J Neuroradiol. (2001) 22:1534–42. 11559501 PMC7974585

[11] Albers GW Marks MP Kemp S Christensen S Tsai JP Ortega-Gutierrez S. Thrombectomy for stroke at 6 to 16 hours with selection by perfusion imaging. N Engl J Med. (2018) 378:708–18. 10.1056/NEJMoa1713973 29364767 PMC6590673

[12] Chung JW Park SH Kim N Kim WJ Park JH Ko Y. Trial of ORG 10172 in acute stroke treatment (TOAST) classification and vascular territory of ischemic stroke lesions diagnosed by diffusion-weighted imaging. J Am Heart Assoc. (2014) 3:1–8. 10.1161/JAHA.114.001119 25112556 PMC4310410

[13] Zaidat OO Yoo AJ Khatri P Tomsick TA Von Kummer R Saver JL. Recommendations on angiographic revascularization grading standards for acute ischemic stroke: a consensus statement and for the cerebral angiographic revascularization, grading (CARG) Collaborators, STIR Revascularization working group, and STIR Thrombol. Stroke. (2013) 44:2630–63. 10.1161/STROKEAHA.113.001972 23920012 PMC4160883

[14] Hacke W Kaste M Fieschi C Von Kummer R Davalos A Meier D. Randomised double-blind placebo-controlled trial of thrombolytic therapy with intravenous alteplase in acute ischaemic stroke (ECASS II). Lancet. (1998) 352:1245–51. 10.1016/S0140-6736(98)08020-9 9788453

[15] Von Kummer R Broderick JP Campbell BCV Demchuk A Goyal M Hill MD. The heidelberg bleeding classification: classification of bleeding events after ischemic stroke and reperfusion therapy. Stroke. (2015) 46:2981–6. 10.1161/STROKEAHA.115.010049 26330447

[16] Rebello LC Haussen DC Belagaje S Anderson A Frankel M Nogueira RG. Endovascular treatment for acute ischemic stroke in the setting of anticoagulation. Stroke. (2015) 46:3536–9. 10.1161/STROKEAHA.115.011285 26470775

[17] Liu M Zheng Y Li G. Safety of recanalization therapy in patients with acute ischemic stroke under anticoagulation: a systematic review and meta-analysis. J Stroke Cerebrovasc Dis. (2018) 27:2296–305. 10.1016/j.jstrokecerebrovasdis.2018.04.012 30017747

[18] Uphaus T Singer OC Berkefeld J Nolte CH Bohner G Niederkorn K. Safety of endovascular treatment in acute stroke patients taking oral anticoagulants. Int J Stroke. (2017) 12:412–5. 10.1177/1747493016677986 28353412

[19] L'Allinec V Sibon I Mazighi M Labreuche J Kyheng M Boissier E. MT in anticoagulated patients: direct oral anticoagulants versus vitamin K antagonists. Neurology. (2020) 94:e842–50. 10.1212/WNL.0000000000008873 31959707

[20] Benavente L Larrosa D García-Cabo C Pérez ÁI Rico M Vega P. Safety and efficacy of mechanical thrombectomy in acute ischemic stroke of anticoagulated patients-a prospective observational study. J Stroke Cerebrovasc Dis. (2016) 25:2093–8. 10.1016/j.jstrokecerebrovasdis.2016.06.006 27378732

[21] Boeckh-Behrens T Schubert M Förschler A Prothmann S Kreiser K Zimmer C. The impact of histological clot composition in embolic stroke. Clin Neuroradiol. (2016) 26:189–97. 10.1007/s00062-014-0347-x 25261075

[22] Zapata-Wainberg G Ximénez-Carrillo Á Trillo S Fuentes B Cruz-Culebras A Aguirre C. Mechanical thrombectomy in orally anticoagulated patients with acute ischemic stroke. J Neurointerv Surg. (2018) 10:834–8. 10.1136/neurintsurg-2017-013504 29275325

