# Supplementary material for: Oral Anticoagulation and Risk of Symptomatic Hemorrhagic Transformation in Stroke Patients Treated With Mechanical Thrombectomy: Data From the Nordictus Registry
Source: Front Neurol. 2020 Nov 26;11:594251. doi: 10.3389/fneur.2020.594251 (PMC7726434; doi:10.3389/fneur.2020.594251)
Supplement: Supplementary file 1 [file Data_Sheet_1.PDF]

Supplementary table 1. Bivariate analysis between procedural characteristics and anticoagulation status.

	All (n=1455)	NON-OAC (n = 1181)	DOACs (n= 81)	VKA (n= 193)	P Value [a]	P Value [b]
Reperfusion, n (%)					**< 0.001**	0.09
IVT bridging EVT	450 (31)	427 (36)	3 (4)	20 (10)		
Primary EVT	1005 (69)	754 (64)	78 (96)	173 (90)		
EVT strategy, Stent retriever, n (%)	804 (58)	667 (599)	37 (47)	101 (54)	0.107	0.48
General anesthesia, n (%)	647 (45)	537 (46)	27 (33)	83 (43)	0.24	0.27
Number of passes					0.64	0.14
1	561 (48)	458 (49)	34 (49)	69 (45)		
2	251 (22)	201 (23)	16 (23)	34 (22)		
>2	274 (23)	220 (24)	16 (23)	38 (20)		
TICI recanalization, n (%)					0.103	0.28
TICI 3	829 (58)	654 (56)	51 (64)	124 (65)		
TICI 2b	407 (28)	346 (30)	21 (26)	40 (21)		

IVT: intravenous thrombolysis, EVT: endovascular treatment, TICI: thrombolysis in cerebral infarction.

[a] P-values indicate comparisons between overall groups.
[b] P-values indicate comparisons between DOACs and VKA.

Supplementary table 2. Logistic regression model: Predictors of mortality at three months of all cohort.

	**OR (IC 95%)**	**P Value**
Age	1.03 (1.02-1.05)	**< 0.001**
Arterial hypertension	1.06 (0.74-1.05)	0.745
Previous or current smoking	1.23 (0.99-1.52)	0.052
Prior mRS	1.37 (1.12-1.68)	**0.002**
Baseline NIHSS	1.07 (1.05-1.1)	**< 0.001**
Vessel occlusion MCA-M1	1.11 (1-1.23)	**0.044**
Atrial fibrillation	0.83 (0.67-1.03)	0.107

mRS: modified Rankin Scale; NIHSS: National Institute of Health Stroke Scale; MCA: Middle cerebral artery.

List of all NORDICTUS investigators

Hospital Clínico Universitario, Valladolid: Mercedes de Lera Alfonso, Javier Reyes-Muñoz, Elisa Cortijo García, Ana I. Calleja, Blanca Talavera de la Esperanza, Isabel Hernández Pérez, Cristina López Sanz, Gonzalo Valle Peñacoba. **Hospital Universitario Central de Asturias:** Sergio Calleja, Lorena Benavente, Carmen García-Cabo, María Rico, Davinia Larrosa, Montserrat González **Hospital Universitario A Coruña:** Alexia Roel, Sabela Cajaraville, María José Feal, María López-Fernández, Guillermo Muñoz. **Hospital Universitario Miguel Servet:** Álvaro Lambea Gil, Agustín Sancho Saldaña, José María Navasa. **Complejo Asistencial Universitario de León:** Ana Fernández Martínez, Sebastián Baldi. **Hospital Universitario de Donostia:** San Sebastián: Maite Martínez Zabaleta, Ana de Arce, Maite Martínez, Félix González, Jon Rodríguez-Antigüedad. José Ángel Larrea, Eñaut Garmendia, Alex Luttich. **Complejo Hospitalario Universitario de Santiago:** Iria López-Dequidt, Iago García-Díaz, Antonio Jesús Mosqueira, José Manuel Pumar. **Hospital Universitario de Burgos:** José María Trejo Gabriel y Galán. Ana Echavarria Íñiguez. **Hospital Universitario Marqués de Valdecilla:** Yésica Jiménez López, José Luis Martín Gurpegui, José Luis Vázquez Higuera, José Ramón Sánchez de la Torre. **Hospital Universitario de Araba:** Vitoria: Jon Segurola Olaizola. **Complejo Hospitalario Universitario de Vigo:** Joaquín Sánchez Herrero. **Hospital San Pedro, La Rioja:** ME Marzo Sola, MA. López-Pérez, M. Gómez-Eguilaz, JM. Pérez-Imbernon, T. Martí Tejada. **Hospital Galdakao-Usansolo, Bizkaia:** Javier Arranz martinez. **Cruces Hospital, Barakaldo:** Moreno-Estebanez A, González E, Fondevila J.
